# Deep learning-based computed tomography urography image analysis for prediction of HER2 status in bladder cancer

**DOI:** 10.7150/jca.101296

**Published:** 2024-10-14

**Authors:** Panpan Jiao, Rui Yang, Yunxun Liu, Shujie Fu, Xiaodong Weng, Zhiyuan Chen, Xiuheng Liu, Qingyuan Zheng

**Affiliations:** 1 Department of Urology, Renmin Hospital of Wuhan University, Wuhan, Hubei, 430060, China.; 2 Institute of Urologic Disease, Renmin Hospital of Wuhan University, Wuhan, Hubei, 430060, China.

**Keywords:** Artificial Intelligence, Deep Learning, Computed Tomography Urography, HER2, Bladder Cancer

## Abstract

**Purpose:** Bladder cancer (BCa) is one of the most common malignant tumors in the urinary system. BCa with HER2 overexpression can benefit from more precise treatments, but HER2 testing is costly and subjective. This study aimed to detect HER2 expression using computed tomography urography (CTU) images.

**Method:** We gathered CTU images from 97 patients with BCa during the excretion phase in Renmin Hospital of Wuhan University, manually delineated the BCa regions, extracted radiomic features using the Pyradiomics package, conducted data dimensionality reduction via principal component analysis (PCA), and trained three models (Logistic Regression [LR], Random Forest [RF] and Multilayer Perceptron [MLP]) to discern the HER2 expression status.

**Results:** Pyradiomics package was used to extract 975 radiological features and the cumulative interpretation area under the variance curve was 90.964 by PCA. Using an MLP-based deep learning model for identifying HER2 overexpression, the area under the curve (AUC) reached 0.79 (95% confidence interval [CI] 0.74-0.86) in the training set and 0.73 (95% CI 0.66-0.77) in the validation set. LR and RF had AUC of 0.69 (95% CI 0.63-0.75) and 0.66 (95% CI 0.61-0.70) in the training set and 0.61 (95% CI 0.55-0.67) and 0.59 (95% CI 0.55-0.63) in the test set, respectively.

**Conclusion:** The study firstly presents a non-invasive method for identifying and detecting HER2 expression in BCa CTU images. It might not only reduce the cost and subjectivity of traditional HER2 testing but also provide a new technical method for the precise treatment of BCa.

## Introduction

Bladder cancer (BCa) is the second most common malignant tumor of the urinary system worldwide^1^. According to the American Cancer Society, it is estimated that there will be 83,190 new cases of BCa and 16,840 new deaths in the United States in 2024^2^. Human epidermal growth factor receptor2 (HER2) primarily encodes a transmembrane tyrosine kinase receptor and is present in various solid tumors. It is associated with angiogenesis and tumorigenesis^3^. Elevated levels of HER2 expression have a clear predictive value for prognosis in gastric cancer^4^. Furthermore, targeted HER2 therapy is a key treatment strategy for many HER2-overexpressing solid tumors, such as breast cancer and colorectal cancer^5^, ^6^.

HER2 is a well-established oncogene involved in the development and progression of BCa. Studies have shown that HER2 overexpression is closely associated with poor prognosis in BCa^7^, ^8^. Research indicates that antibody-drug conjugates targeting HER2 have demonstrated significant clinical efficacy in treating HER2-overexpressing locally advanced or metastatic urothelial carcinoma^9^, ^10^. Furthermore, combining these antibody-drug conjugates with immune checkpoint inhibitors for the neoadjuvant treatment of urothelial carcinoma has shown promising preliminary results^11^.

Immunohistochemistry (IHC) staining is commonly used to evaluate HER2 status at the protein level, while fluorescence in situ hybridization uses DNA probes to detect HER2 status^12^, ^13^. Currently, IHC staining for HER2 is a key component of postoperative pathological examinations and management of BCa in clinical practice. However, IHC staining significantly increases both the time and financial burden on patients. Additionally, due to the high heterogeneity of HER2 expression in solid tumors like BCa, histological examination of postoperative specimens and biopsy tissues may not fully capture the entire tumor. Consequently, patients with HER2 overexpression who present as false negatives might experience delayed or missed treatment opportunities^14^. Moreover, despite the existence of consensus guidelines for interpreting HER2 IHC staining, the interpretation relies entirely on the pathologist's expertise^15^. Therefore, rapid diagnosis and proper treatment of patients with HER2-overexpressing BCa are crucial for extending their lifespan and improving their quality of life.

In this study, we aimed to directly predict HER2 expression from computed tomography urography (CTU) images of BCa patients. This approach could substantially alleviate the comprehensive costs for patients and yield more potential benefits, while also significantly easing the workload of pathologists. To our knowledge, this is the first endeavor to predict HER2 expression directly from CTU images.

## Methods

**Figure 1:**
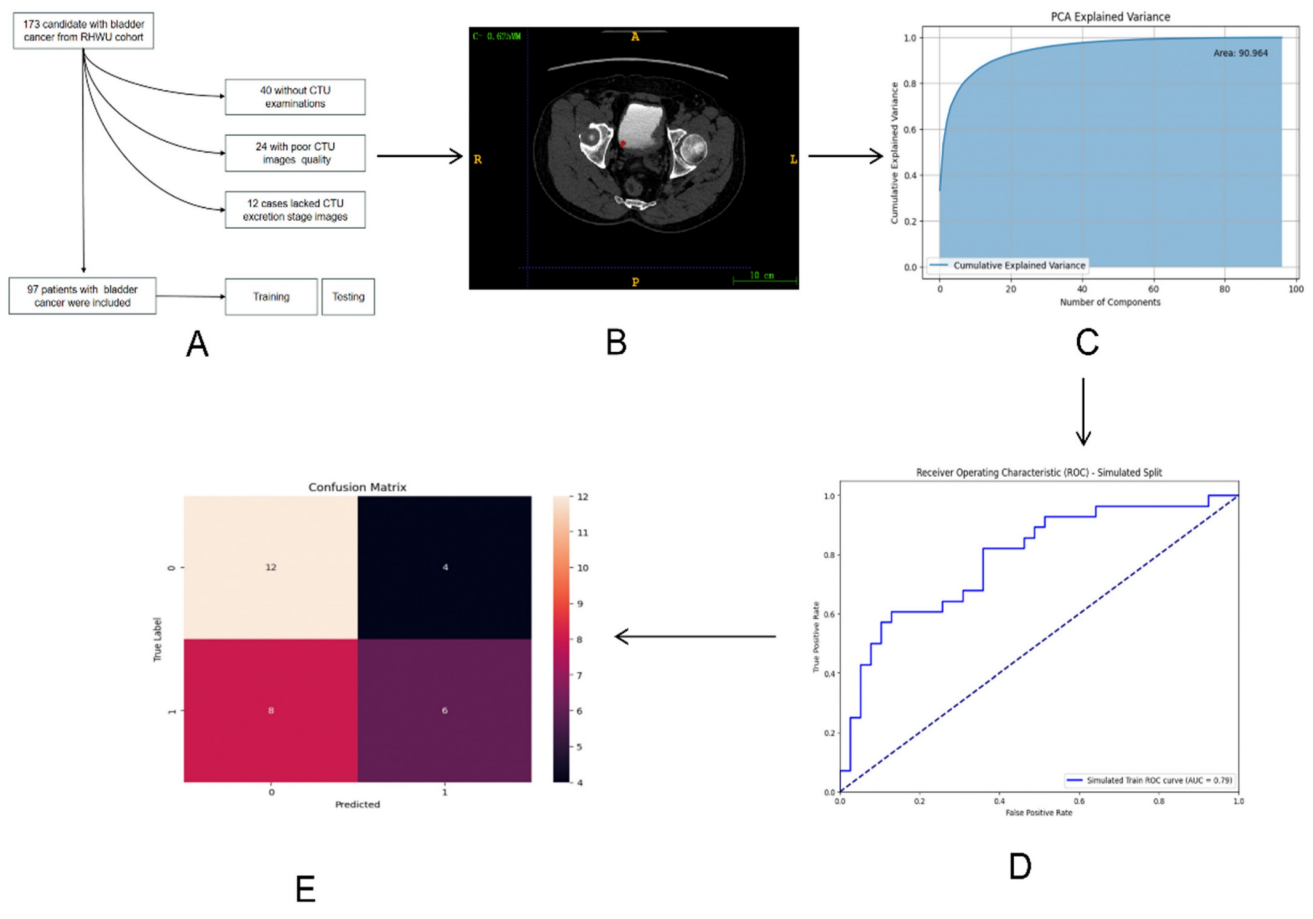
The whole process of the research workflow is shown. (A) Establishment of RHWU cohort; (B) Mapping ROI areas and extract radiomics features; (C) Data dimensionality was reduced by principal component analysis (PCA); (D) ROC curve was drawn to compare the performance of each model; (E) Model performance evaluation.

The entire design process of this study is depicted in Figure 1.

### Study population

**Figure 2:**
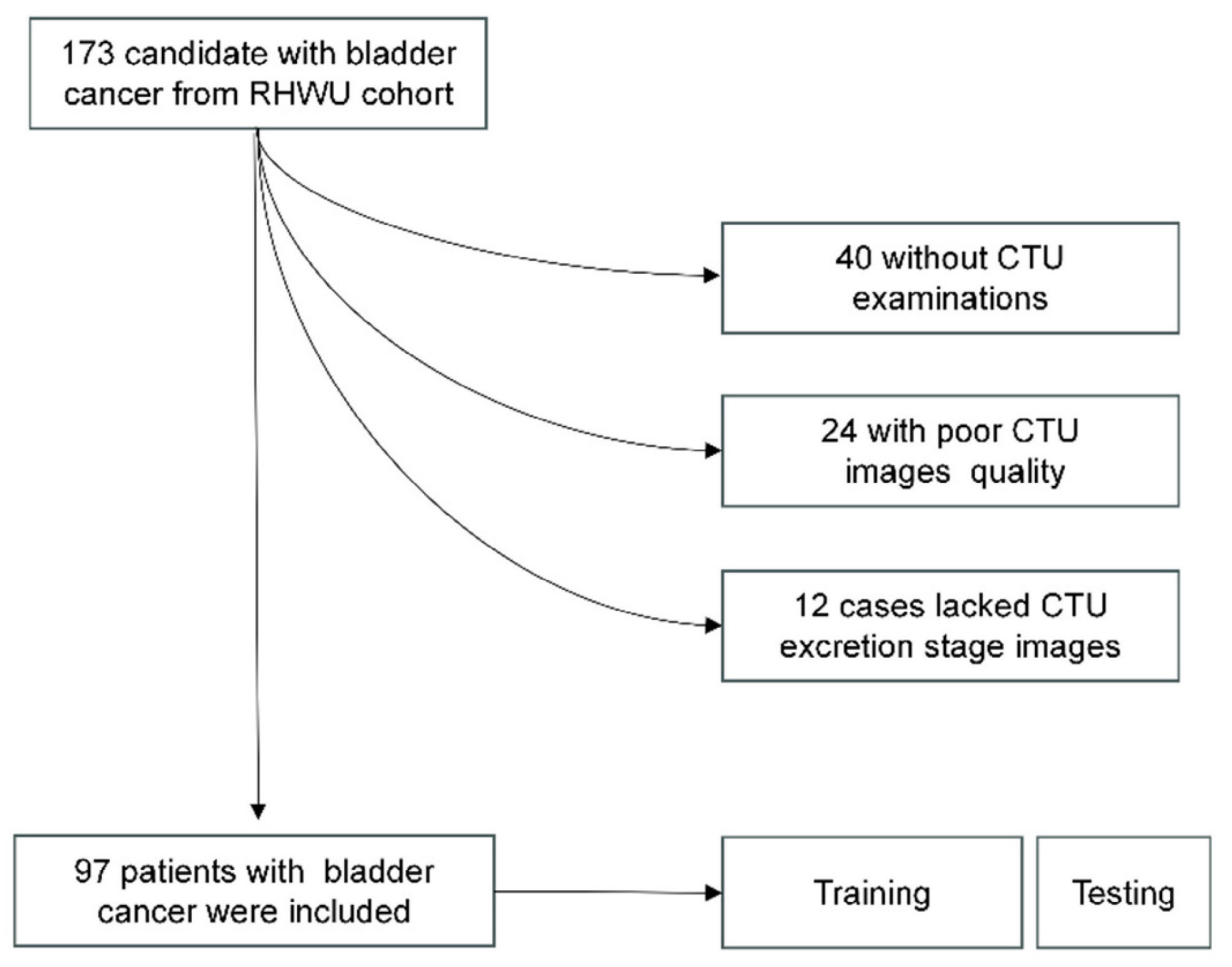
Description of pathways to recruit patient from the RHWU cohort.

This study adhered to the Helsinki Declaration and obtained approval from the Clinical Research Ethics Committee, Renmin Hospital of Wuhan University (RHWU; Wuhan, Hubei, China). All participants provided informed consent (Ethical Approval Number: WDRY2022-K077). The study recruited 173 patients who underwent bladder tumor surgery in RHWU, from 2017 to 2023. Patients meeting the following criteria were excluded from the study: 12 patients lacked CTU data during the secretion period, 40 patients lacked CTU data, and 24 patients had poor-quality CTU images during the secretion period. These patients were all excluded. We ultimately included 97 patients in RHWU cohort. Only cases with a confirmed pathological diagnosis of BCa, no history of other cancers, known HER2 status, no history of targeted or immunotherapy, and clinical data such as age, gender, T stage, lymphovascular invasion, histological grade, etc., were included. The patient recruitment process is illustrated in Figure 2.

### HER2 status assessment

Immunohistochemistry staining was employed to determine the HER2 status, with scoring based on the current ASCO/College of American Pathologists guidelines for scoring HER2 in gastric cancer^13^, ^16^, ^17^. An IHC staining score of 3+ or 2+ was deemed positive, while patients with scores of 0 or 1+ were classified as negative. The examples of HER2 expression status as positive and negative are found in Figure S1.

### Image analysis

We gathered CTU images with a slice thickness of 5 mm from patients diagnosed with BCa, known HER2 expression status, and no prior targeted therapy. The BCa imaging data collected underwent independent and randomized evaluation by two radiologists, each with 10 years of experience, who were blinded to the clinical data. Following the consolidation of their reports, a third senior radiologist with 15 years of experience reviewed the CTU images and made the final decisions. The images during the excretion phase were ultimately recorded as digital imaging data and communications in medicine (DICOM) files.

### BCa lesion segmentation and radiomic feature extraction

**Figure 3:**
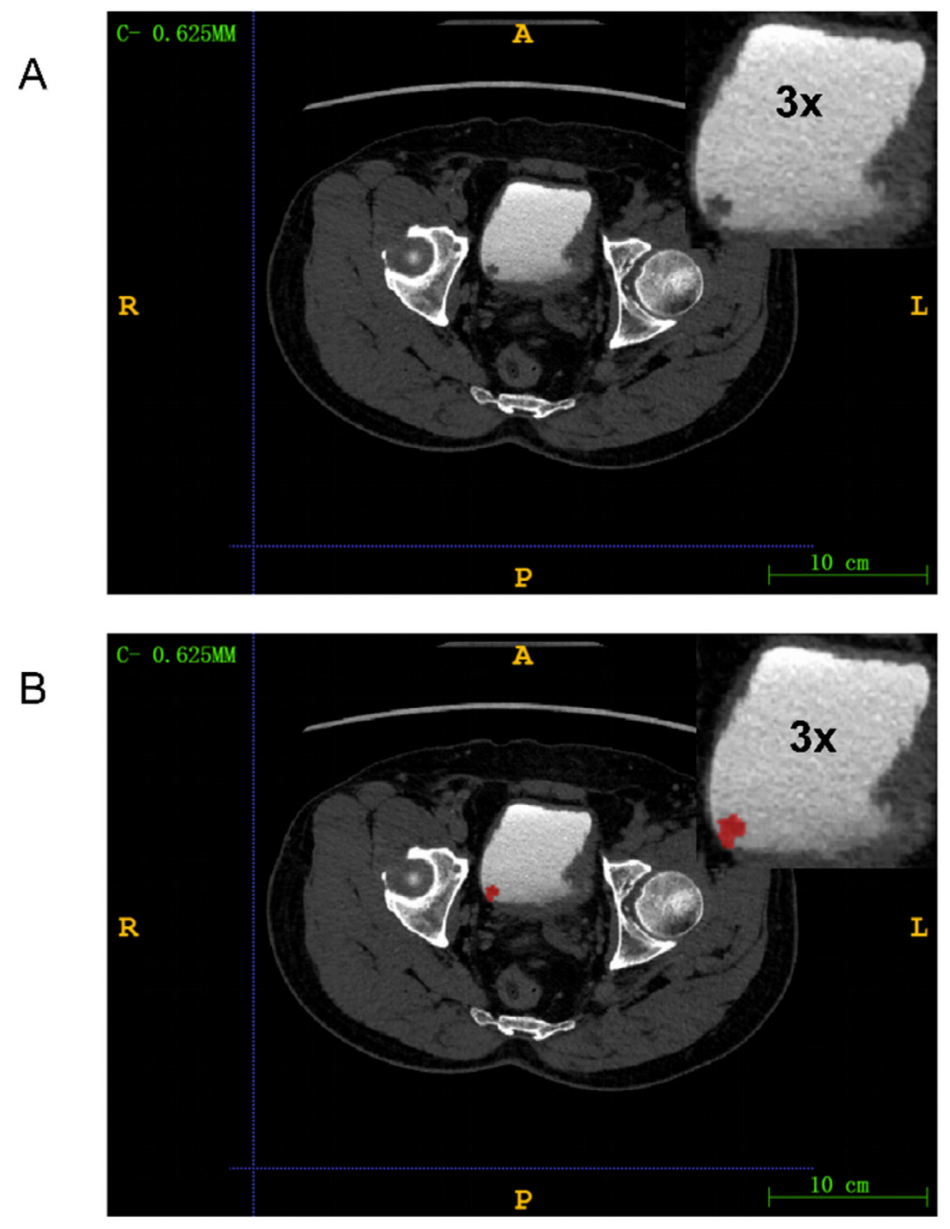
** Mapping ROI areas.** (A) Imaging information of bladder cancer; (B) Outline of bladder cancer: Red region is bladder cancer. The upper right corner part is an enlarged image of the bladder tumor by three times.

We used ITK-SNAP (version 3.8.0) to manually delineate regions of interests (ROIs) around lesions in DICOM images. A radiologist with 15 years of experience reviewed and confirmed the manually delineated ROIs to ensure they encompassed tumor tissue for radiomic feature extraction (Figure 3). The medical images in DICOM (.dcm) format were then converted to NIfTI (.nii) format.

We used Pyradiomics (version 3.1.0) to extract 975 radiomic features from the ROIs. (Table S1) This package is available on GitHub^18^. The extracted features included shape features, first-order statistics, gray level co-occurrence matrix (GLCM), gray level run length matrix (GLRLM), gray level size zone matrix (GLSZM), and gray level dependence matrix (GLDM). The radiomic features were automatically extracted by the Pyradiomics package and saved as comma-separated values files. The example of HER2 expression being negative and positive in CTU images can be found in Figure S2.

### Model development

Principal component analysis (PCA) was used to determine the extracted and normalized radiomic features. The lines in the graph represented the cumulative proportion of variance explained. Each point indicated the proportion of data variance explained by adding one principal component. The point where the curve began to flatten indicated that additional principal components would not significantly increase the explained variance. By selecting the point just before the curve flattens, we could achieve dimensionality reduction while retaining most of the information.

We developed three models: two machine learning models based on Random Forest (RF) and Logistic Regression (LR), and a deep learning (DL) model for HER2 prediction using a Multi-Layer Perceptron (MLP). RF performed classification tasks through ensemble learning and random feature selection, while LR was a widely used linear model that classified using the Sigmoid function and log-odds. First, we evaluated the models on the training set, followed by validation on the validation set. To assess the models' performance in predicting HER2 expression status, we plotted receiver operating characteristic (ROC) curves and quantified prediction accuracy using the area under the curve (AUC). Additionally, we evaluated the models' performance using confusion matrices, learning curves, and precision-recall curves.

### Statistical analysis

We divided 7/10 of the patients into the training dataset and 3/10 into the testing dataset using computer-generated random numbers. Data visualization was performed using R (version 3.2.0). We used Wilcoxon rank sum test, Fisher's exact test, and Pearson's Chi-squared test to compare baseline information between the training and validation cohorts. A p-value less than 0.05 (two-sided) was considered statistically significant.

## Results

### Patient characteristics

The complete baseline information for the RHWU cohort is summarized in Table 1.

The baseline information for both the training and validation sets is summarized in Table 2. There were no significant statistical differences observed between the training and testing sets regarding age, gender, AJCC 8th bladder tumor staging, and other factors.

### PCA results

**Figure 4:**
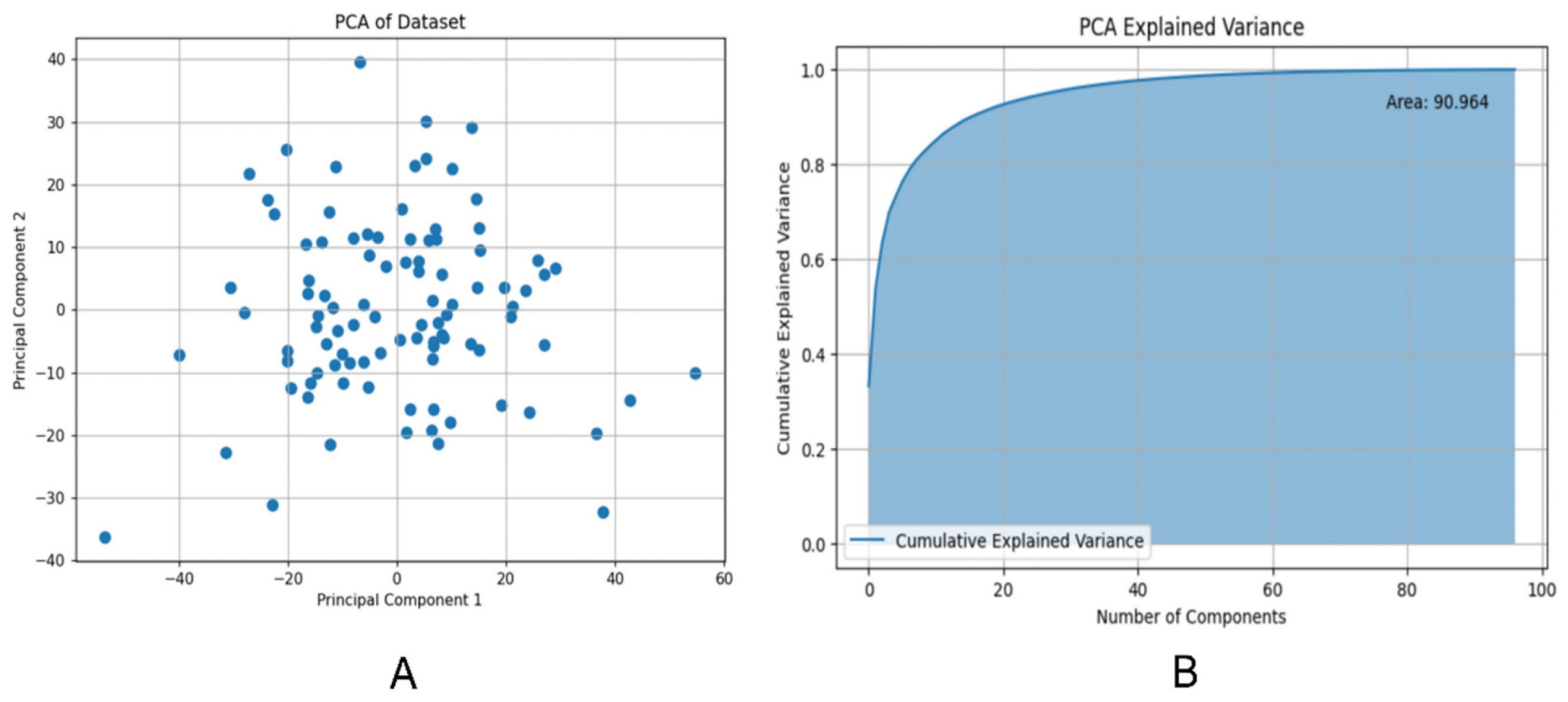
(A) Projection of principal component 1 and 2 in two dimensions; (B) Cumulative explanatory variance curve of principal component analysis (PCA) method.

We employed PCA to reduce the dimensionality of the feature data. Figure 4A demonstrated the outcomes of this dimensionality reduction process conducted through PCA. In particular, the figure depicted a two-dimensional scatter plot illustrating the projection of the data onto the first two principal components. The horizontal axis represented PC1, and the vertical axis represented PC2. Each point on the plot symbolized the projection of an original data point onto these two principal component axes. PC1 corresponded to the axis that maximized the variance of the data, while PC2 was orthogonal to PC1 and maximized the remaining variance. These principal components, being linearly independent, captured the most significant directions of variation in the data.

Figure 4B presented the Cumulative Explained Variance curve of the PCA model. On the horizontal axis, we had the number of components, while the vertical axis represented the cumulative explained variance. The blue area in the graph illustrated the cumulative explained variance. This cumulative explained variance indicated the proportion of total variance in the original data as we increased the number of principal components. As depicted in the graph, with an increasing number of components, the cumulative explained variance gradually rises and eventually converges to 1 (i.e., 100%). This indicated that by incorporating a sufficient number of components, we could account for all the variance in the original data. Particularly noteworthy was the rapid increase in cumulative explained variance when the number of principal components was relatively low (e.g., the first 20 components), suggesting that these initial components captured the majority of the variance in the original data. However, as the number of components continued to increase, the rate of increase in cumulative explained variance diminished, ultimately reaching a plateau. This suggested that additional components contributed progressively less to explaining the total variance. The area under the Cumulative Explained Variance curve was 90.964, indicating that approximately 90.964% of the original data's variance could be explained by the first 100 components.

### The performance of the three models

**Figure 5:**
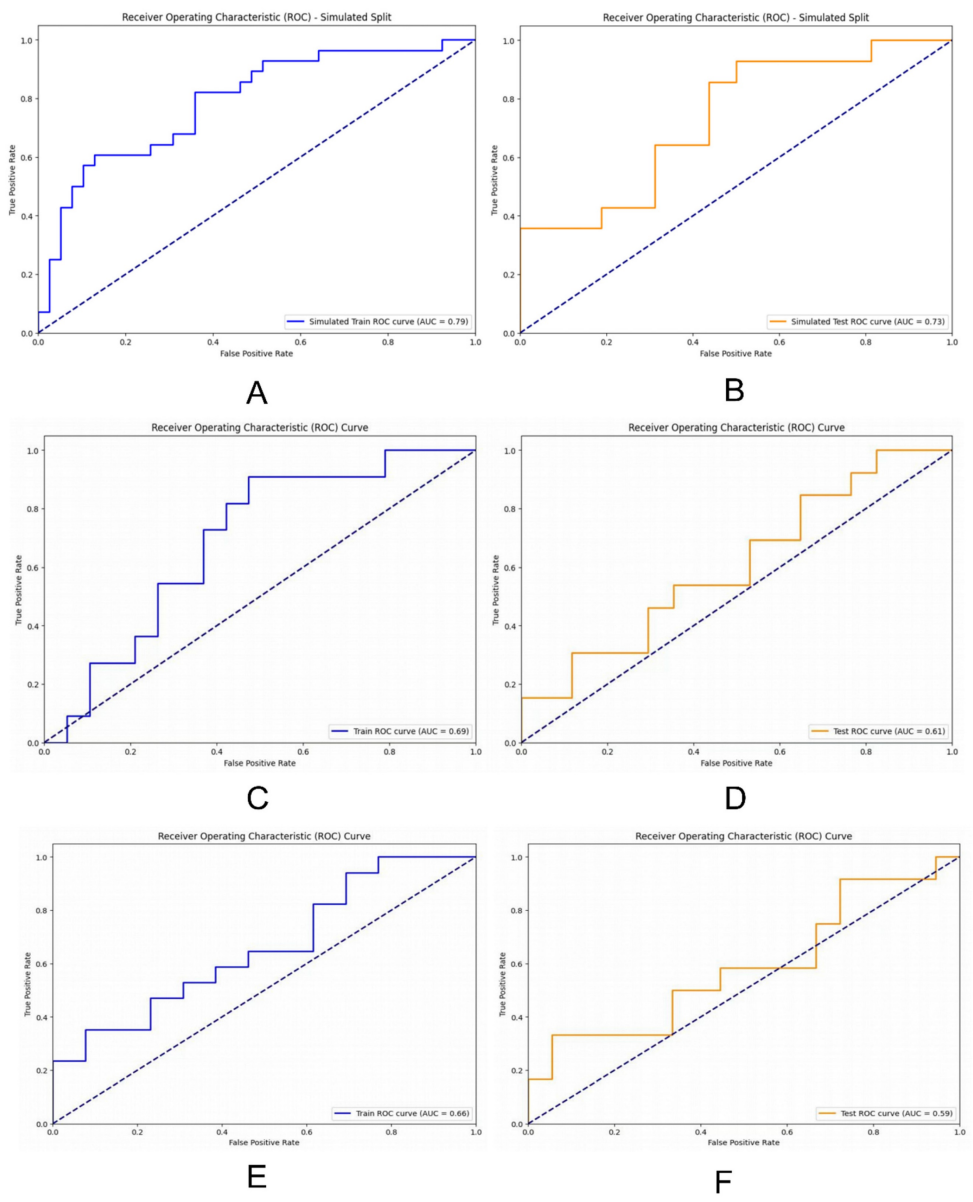
ROC curves were drawn to compare the performance of each model; **(A) and (B)** ROC curves were used to describe the performance of deep learning model based on multi-layer perceptrons (MLP) in training sets and testing sets; **(C) and (D)** ROC curves were used to describe the performance of RF model in training sets and testing sets; **(E) and (F)** ROC curves were used to describe the performance of LR model in training sets and testing sets.

We assessed our models' performance by plotting ROC curves and comparing their performance using the AUC, where a higher AUC indicated better performance. The DL model trained with MLP exhibited strong performance on both the training and validation sets, with an AUC of 0.79 (95% CI 0.74-0.86) on the training set and 0.73 (95% CI 0.66-0.77) on the validation set. The RF model achieved an AUC of 0.69 (95% CI 0.63-0.75) on the training set and 0.61 (95% CI 0.55-0.67) on the validation set. Conversely, the LR model achieved an AUC of 0.66 (95% CI 0.61-0.70) on the training set and 0.59 (95% CI 0.55-0.63) on the validation set (Figure 5).

### Performance evaluation of the DL model based on MLP

**Figure 6:**
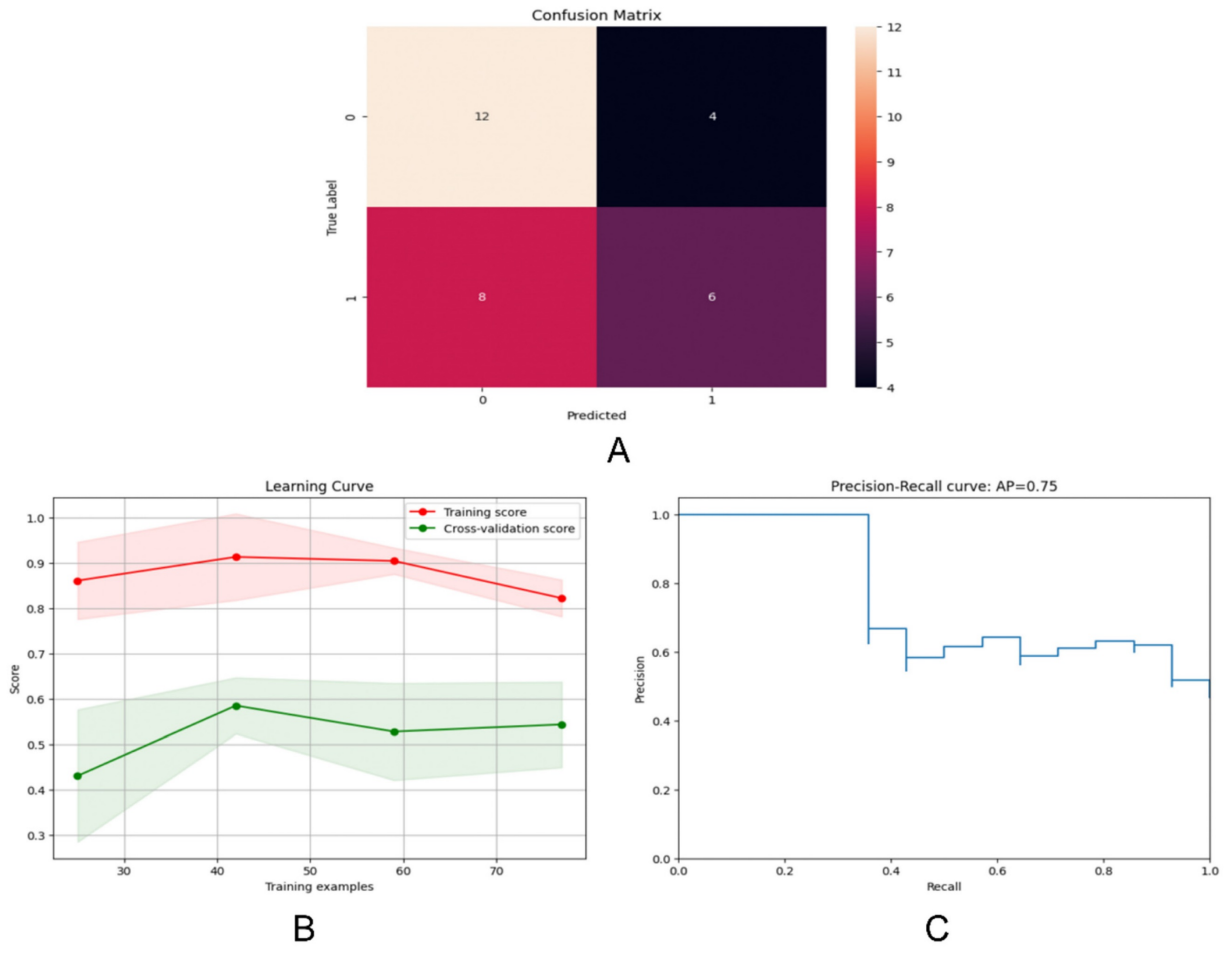
(A)The confusion matrices for the DL model; (B) The learning curve for the training set; (C) The Precision-Recall curve for the DL model.

We assessed the model's performance using a confusion matrix, which compared the model's predicted outcomes with the actual results. (Figure 6A) Each row represented the true labels, while each column represented the predicted labels. We observed that the model performed well for class 0 (Her2-positive), with a high number of true positives (12) but some false positives (4).

Examining the learning curve, we noticed that the training score consistently remained high as the amount of training data increased. (Figure 6B) This suggested that the model effectively learned from more data, thereby improving its performance. Similarly, as the amount of training data increased, the cross-validation score also improved, indicating enhanced performance on unseen data. Interestingly, we observed that the gap between the training score and the cross-validation score gradually diminished with the increase in training sample size, indicating robust generalization of the model.

The precision-recall curve is a valuable tool for evaluating classification models, especially in binary classification scenarios (Figure 6C). It illustrates the relationship between Precision and Recall across different thresholds. Notably, the model achieved an average precision of 0.75.

## Discussion

As far as we know, this is the first imaging genomics model to predict HER2 overexpression in BCa tissue directly from CTU images. Serving as a non-invasive imaging biomarker for HER2 overexpression in BCa patients, it demonstrated excellent discrimination of HER2 expression status in both the training and validation sets.

Radiomics enables the high-throughput extraction of imperceptible information from medical imaging, facilitating automated quantitative analysis that comprehensively quantifies the heterogeneity of tumors. It provides detailed insights into the microstructure and biological characteristics of tumors.

Previously, numerous studies have been published on DL models for detecting HER2 overexpression in other solid tumors. S. Farahmand *et al.* proposed a convolutional neural network approach that achieved HER2 identification on H&E-stained whole slide images with an AUC of 0.9^19^. Xiao Guan *et al.* utilized Predictionnet, based on Vision Transformer, to predict HER2 expression status in enhanced CT datasets from The Cancer Imaging Archive gastric cancer dataset, achieving an AUC of 0.9721^20^. Z. Xu *et al.* established a 3-block-DenseNet-based DL model for predicting HER2 expression status in ultrasound images of breast cancer, achieving an accuracy of 85.79% and an AUC of 0.87 in the training set^21^.

We utilized a MLP-based model to analyze the expression status of HER2 in BCa. MLP, a type of feedforward artificial neural network, comprises input layers, hidden layers, and output layers. It is commonly employed to solve classification and regression problems by learning the mapping relationship between inputs and outputs from large amounts of training data.

PCA is advantageous in removing redundant information and noise from the data, thereby simplifying the model and enhancing computational efficiency. On the other hand, MLP demonstrates strong nonlinear fitting ability, making it suitable for various tasks such as classification and regression. By learning feature representations in the hidden layers, MLP can automatically discover abstract features and patterns in the data, improving the model's generalization ability. Moreover, MLP exhibits some robustness to noise and outliers to a certain extent, and its robustness can be further enhanced through regularization and other techniques.

The model serves to identify patients with false-negative HER2 overexpression due to tumor heterogeneity. By discerning HER2 expression status in BCa through CTU images, it alleviates the overall burden on patients. This novel model has the potential to identify patients who are likely to benefit from targeted therapies, thereby streamlining the medical diagnostic and treatment processes.

Our study has several limitations. First, as a single-center retrospective study with a relatively small sample size, the generalizability of the model may be limited. Second, the HER2 status determined through specimen testing might not accurately represent the true expression of HER2 overexpression in BCa, potentially leading to discrepancies. Third, the complexity and inconsistency of CTU operations may affect the stability and reproducibility of the model. Lastly, the supervised classification algorithm we used is based on the deep learning model of bladder cancer CTU images, which warrants further research and may offer higher predictive accuracy. We aim to conduct multi-center prospective multi-omics studies in the future to enhance the overall performance and generalizability of the DL model.

## Conclusion

The study developed a DL model that could predict the HER2 expression status in BCa patients using CTU images. The results from this model could serve as a non-invasive imaging biomarker to screen patients who are suitable for anti-HER2 targeted therapy.

## Supplementary Material

Supplementary figures and table.

## Figures and Tables

**Table 1:** Clinical, biological and pathological characteristics of bladder cancer patients included in the RHWU cohort.

	RHWU (*N* = 97)
Age (years)	68.20±1.03
Gender	
Female	10 (10.31%)
Male	87 (89.69%)
pT stage	
pTis	3 (3.09%)
pTa	43 (44.33%)
pT1	20 (20.62%)
pT2	24 (24.74%)
pT3	5 (5.15%)
pT4	2 (2.06%)
pN stage	
pN0	91 (93.81%)
pN1-3	6 (6.19%)
pM stage	
pM0	97 (100.00%)
pM1	0 (0%)
pMx	0 (0%)
pTNM stage	
Stage 0a	43 (44.33%)
Stage 0is	3 (3.09%)
Stage I	20 (20.62%)
Stage II	24 (24.74%)
Stage III	7 (7.22%)
Stage IV	0 (0%)
Lymphovascular invasion	
No	66 (68.04%)
Yes	14 (14.43%)
Missing	17 (17.53%)

**Table 2:** Clinical, biological and pathological characteristics of bladder cancer patients in the primary and validation cohorts.

Variable	Overall, N = 97^1^	Training group, N = 67^1^	Testing group, N = 30^1^	p-value^2^
Age	69 (62, 76)	69 (64, 77)	68 (60, 72)	0.13
Gender				>0.99
Female	10 (10%)	7 (10%)	3 (10%)	
Male	87 (90%)	60 (90%)	27 (90%)	
HER2				0.077
HER2(-)	55 (57%)	34 (51%)	21 (70%)	
HER2(+)	42 (43%)	33 (49%)	9 (30%)	
AJCC stage				0.70
0a	43 (44%)	31 (46%)	12 (40%)	
0is	3 (3.1%)	1 (1.5%)	2 (6.7%)	
I	20 (21%)	13 (19%)	7 (23%)	
II	24 (25%)	17 (25%)	7 (23%)	
IIIA	2 (2.1%)	2 (3.0%)	0 (0%)	
IIIB	5 (5.2%)	3 (4.5%)	2 (6.7%)	

^1^Median (IQR) or Frequency (%) ^2^Wilcoxon rank sum test; Fisher's exact test; Pearson's Chi-squared test
