# Supplementary material for: Deep learning-based computed tomography urography image analysis for prediction of HER2 status in bladder cancer
Source: J Cancer. 2024 Oct 14;15(19):6336–44. doi: 10.7150/jca.101296 (PMC11540498; doi:10.7150/jca.101296)
Supplement: Supplementary file 1 — Supplementary figures and table. [file jcav15p6336s1.zip › Supplementary materials.pdf]

Supplementary Information

Figure S1. Immunohistochemical staining is used for HER2 assessment. Figure A is an image of bladder cancer tissue after immunohistochemical staining showing a positive expression of HER2. Figure B is a 20x magnified image of the local area of Figure A, with HER2 expression remaining positive. Figure C is an image of bladder cancer tissue after immunohistochemical staining showing a negative expression of HER2. Figure D is a 20x magnified image of the local area of Figure C, with HER2 expression remaining negative.

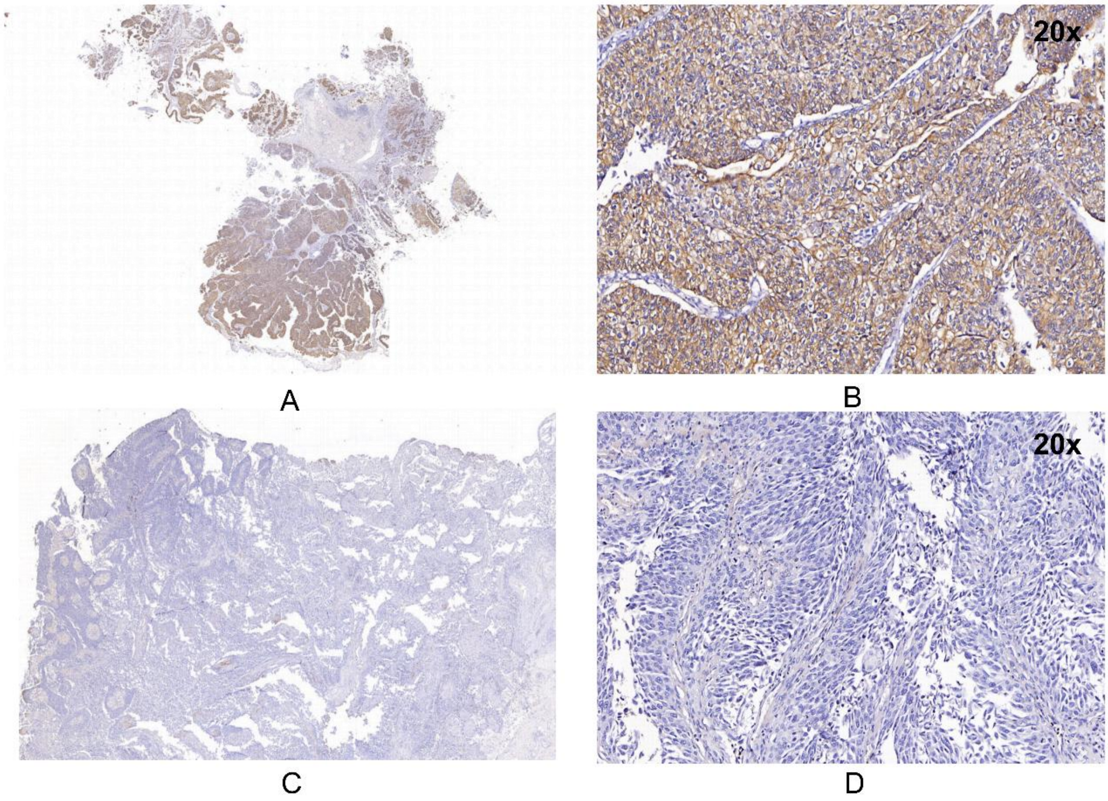

Figure S2. The presentation of HER2 expression status in CTU images. Figure A shows the CTU image with positive HER2 expression, and Figure B shows the CTU image with negative HER2 expression. The bladder tumor is indicated by the red circle.

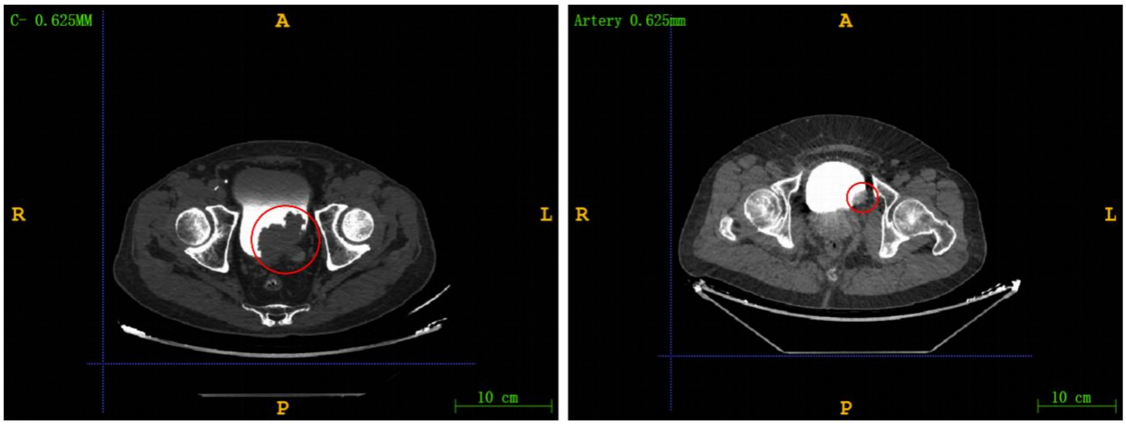

A B

Table S1. Radiomic features can be found in excel.
